# Biotechnological Applications of C-Type Lectins Isolated from Snake Venoms

**DOI:** 10.3390/molecules31111906

**Published:** 2026-06-01

**Authors:** Ellynes Amancio Correia Nunes, Geovanna Moura, Breno Emanuel Farias Frihling, Juliana Ferreira de Lima, Adriel Parahyba Lacerda, Rayane Vasconcelos, Ana Paula de Araújo Boleti, Ana Cristina Jacobowski, Juliana Zuliani, Elizeu Antunes dos Santos, Hector Koolen, Karla Luna, Maria Ligia Rodrigues de Macedo, Ludovico Migliolo

**Affiliations:** 1 Programa de Pós-Graduação em Bioquímica e Biologia Molecular, Universidade Federal do Rio Grande do Norte, Natal 59.078-970, RN, Brazil; ellynesnunes@gmail.com (E.A.C.N.); geovanna_m3@hotmail.com (G.M.); rgabrielle27@gmail.com (R.V.); elizeu.ufrn@gmail.com (E.A.d.S.); 2 S-Inova Biotech, Program de Pós-Graduação em Biotecnologia, Universidade Católica Dom Bosco, Campo Grande 79.117-900, MS, Brazil; brenoemanuelfarias@gmail.com (B.E.F.F.); adrielparhyba@gmail.com (A.P.L.); apboleti@gmail.com (A.P.d.A.B.); 3 Faculdade de Ciências Farmacêuticas, Alimentos e Nutrição, Universidade Federal do Mato Grosso do Sul, Campo Grande 79.070-900, MS, Brazil; ligiamacedo18@gmail.com; 4 Laboratório de Imunologia Celular Aplicada a Saúde, Fundação Oswaldo Cruz, Porto Velho 76.812-245, RO, Brazil; julianaferreria15@gmail.com (J.F.d.L.); zuliani.juliana@gmail.com (J.Z.); 5 Programa de Pós-Graduação em Saúde e Desenvolvimento na Região Centro-Oeste, Universidade Federal do Mato Grosso do Sul, Campo Grande 79.070-900, MS, Brazil; anacristinaj@gmail.com; 6 Centro Multiusuário para Análise de Fenômenos Biomédicos, Universidade Estadual do Amazonas, Manaus 69.065-001, AM, Brazil; hkoolen@uea.edu.br; 7 Centro de Ciências Biológicas e da Saúde, Universidade Federal de Campina Grande, Campina Grande 58.429-900, PB, Brazil; karlaceatox@yahoo.com.br

**Keywords:** serpent, toxins, proteins, antitumor, agglutination, angiogenesis

## Abstract

Snake venoms are rich sources of molecules with pharmacological potential, with approximately 90% of their composition consisting of proteins and peptides responsible for their biological activities. These proteins are classified as enzymatic or non-enzymatic. Enzymatic proteins function as catalysts in regulatory chemical reactions, whereas non-enzymatic proteins, despite lacking catalytic activity, play essential roles in physiological processes. Lectins are non-enzymatic proteins of non-immune origin characterized by carbohydrate- and glycoprotein-binding domains, enabling their ability to agglutinate erythrocytes. C-type lectins and C-type lectin-like proteins are commonly found in snake venoms and are associated with hemostatic disturbances, particularly bleeding and coagulation disorders. This review provides a comprehensive analysis of studies published over the past decade on lectins isolated from snake venom, addressing their definitions, classifications, structural characteristics, and mechanisms of action, as well as their relevance in biotechnological applications. Although progress has been made in elucidating their pharmacological properties, most studies have focused on plant lectins. In contrast, research on snake venom lectins remains limited, particularly regarding their heterologous activities. This gap, especially compared to other venom-derived molecules, highlights the need to further expand research on this class of proteins.

## 1. Introduction

Snake venoms consist of a molecular complex with about 90% proteins and peptides in their composition. These proteins can be enzymatic or non-enzymatic and are responsible for the characterization of clinical aspects observed in snakebite envenomation. The main groups of toxins of medical and scientific importance can be classified as neurotoxins, which act on the central and peripheral nervous systems, and proteins that promote muscle toxicities and hemostatic changes, such as metalloproteinases, phospholipases, disintegrins, peptides, and C-type lectins [1,2].

Lectins, which present a binding site for carbohydrates and carbon-binding glycoproteins, are non-enzymatic proteins of non-immune origin that naturally agglutinate erythrocytes and are found in all living organisms, including snake venoms. Due to their non-immunological origin, these proteins differ from other molecules with carbohydrate-binding sites and, unlike immunoglobulins, have two or more binding sites capable of interacting reversibly with carbohydrates [1,3,4]. Therefore, one of the most common ways of classifying or grouping lectins is based on the group of organisms in which they are identified, such as plant lectins, fungal lectins, animal lectins, and bacterial lectins [5]. Moreover, these proteins can also be grouped according to their carbohydrate-binding specificity, such as galactose-binding lectins, mannose-binding lectins, lactose-binding lectins, etc [6,7].

C-type lectins (CTLs) are defined by their Ca^2+^-dependent carbohydrate-binding activity, governed by a highly conserved Carbohydrate Recognition Domain (CRD). The ligand specificity of the CRD is primarily determined by tripeptide motifs: the EPN (Glu-Pro-Asn) motif typically coordinates mannose, glucose, and N-acetylglucosamine (GlcNAc), whereas the QPD (Gln-Pro-Asp) motif is associated with galactose and GalNAc recognition. Furthermore, the conserved WND (Trp-Asn-Asp) sequence is essential for the stabilization of the primary Ca^2+^-binding site, and WIGL (Trp-Iso-Gly-Leu) assists with the formation and structural stability of lectin domains [4,8,9].

As the largest group of lectins identified and studied in animals, CTLs have been widely investigated using molecular and bioinformatic approaches [10,11]. These studies have enabled their classification into various subgroups based on sequence similarities, suggesting a shared evolutionary origin [12,13]. Although snake venom CTLs have a carbohydrate-binding site, binding is not mandatory, nor is the recognition of Ca^2+^. It is important to note that many proteins with activities like CTLs have been described in snake venoms, exhibiting various heterologous activities related to hemostatic disorders triggered by the group, such as antitumor, antithrombotic, and antiangiogenic activities, as well as antimicrobial activities [13,14]. In the venom of some snake species (Figure S1), such as *Crotalus durissus* and *Bothrops jararacussu*, for example, CTLs and C-type lectin-like proteins (snaclecs) have been identified [15,16]. These proteins differ both structurally and functionally from classical C-type lectins, as they lack lectin activity despite exhibiting structural similarities [17]. Snaclecs display a broad range of biological functions, particularly in the modulation of hemostasis [18]. They may function as anticoagulants or procoagulants, and as agonists or antagonists of platelet activation, thereby influencing various components of the coagulation cascade and platelet function [19,20,21].

Regarding their structure, CTLs consist of two homologous subunits, the α subunit (A chain) and the β subunit (B chain), typically formed as heterodimers or oligomers of heterodimers, while classic CLT lectins, derived from snake venom, are composed of homodimers or homoligomers [4]. Due to their selective action on different physiological targets, this group of non-enzymatic proteins has been gaining prominence in biotechnology research, thereby demonstrating their great potential to aid in and propose new therapies for various diseases that currently pose serious public health problems worldwide [12]. To provide a contemporary overview of these emerging biomedical applications, this review specifically focuses on literature published over the past decade, ensuring a targeted discussion on the most recent advances in lectin research.

## 2. Lectin Properties

In the evolutionary landscape of viperid and elapid venoms, CTLs represent a paramount example of functional exaptation and molecular diversification. Recent phylogenomic analyses led by Bryan Fry demonstrate that these non-enzymatic proteins, particularly the heterodimeric ‘snaclecs,’ have evolved through a process of accelerated gene duplication and focal mutagenesis within the CTL domain [22]. This evolutionary trajectory has enabled the transition from ancestral Ca^2+^-dependent carbohydrate binding to the high-affinity targeting of key hemostatic components, such as platelet receptors glycoprotein lb (GPlb) and glycoprotein VI (GPVI), and coagulation factors (IX, X). By forming complex quaternary structures, these toxins exert potent anticoagulation or procoagulation effects, circumventing the physiological defenses of prey through a dynamic ‘red queen’ arms race. Consequently, the structural plasticity of the CTL scaffold not only underscores the biochemical versatility of snake venoms but also highlights their significance as refined tools for studying vascular biology and developing novel therapeutic anticoagulants [23].

This specialized toxicological role is anchored in the fundamental biochemical architecture of the lectin family, which dictates how these proteins interface with biological membranes and cellular receptors. Lectins are di- or multivalent carbohydrate-binding proteins characterized by at least one carbohydrate recognition domain (CRD) that facilitates synergistic binding with specific ligands [3,7]. A hallmark of these proteins is their ability to cross-link glycoconjugates on erythrocyte membranes, a property traditionally utilized in hemagglutination assays to determine blood-type specificity and inhibitory carbohydrate motifs. Notably, the ability of lectins to selectively agglutinate specific ABO blood groups is determined by their fine specificity for distinct surface glycan structures. This process occurs when these proteins recognize and bind to specific terminal sugar residues, such as N-acetylgalactosamine for the A antigen or galactose for the B antigen, present on the erythrocyte membrane [24,25,26,27]. Beyond simple binding, lectin specificity and structural integrity are governed by highly conserved amino acid motifs, the presence of a signal peptide for secretory pathway targeting, and conserved cysteine residues essential for disulfide bond formation (Figure S1) and stabilized by non-covalent forces, including hydrogen bonds and Van der Waals interactions [13,28,29]. In many CTLs, divalent metal ions (notably Ca^2+^) are essential for maintaining the structural conformation required for ligand docking [30].

In animals, lectins are ubiquitously expressed from invertebrates to humans, generally categorized as either integral membrane receptors or soluble proteins [31,32]. While historically classified by their source, they are now more precisely grouped by structural homology. For instance, CTLs are subdivided into 17 distinct groups, including lecticans, selectins, and collectins, based on their domain architecture [8]. Beyond their structural roles, lectins are fundamentally recognized as key components of the innate immune system across diverse taxa [33]. In most well-studied organisms, these proteins function as pattern recognition receptors that identify specific carbohydrate moieties on the surfaces of pathogens, such as bacteria, fungi, and protozoa [34,35]. In vertebrates, this recognition is intricately linked to downstream immune responses, including opsonization and complement activation, underscoring their essential role in host defense mechanisms [36].

## 3. C-Type Lectins (CTLs) and Snake C-Type Lectin-like Proteins (Snaclecs)

CTLs are defined by their Ca^2+^-dependent CRDs, though some family members have evolved Ca^2+^-independent mechanisms, expanding their functional repertoire [8,21]. In toxinology, snake venom CTLs are categorized into “true” CTLs and C-type lectin-like proteins (Snaclecs). These molecules are significant research targets due to their multifaceted roles in modulating hemostasis, inflammation, and potential antitumor [37,38,39] pathways. The primary distinction between classical CTLs and snaclecs lies in their structural dependence on Ca^2+^ for ligand interaction rather than a strict requirement for carbohydrate binding [4,40].

### 3.1. True C-Type Lectins

**Figure 1:**
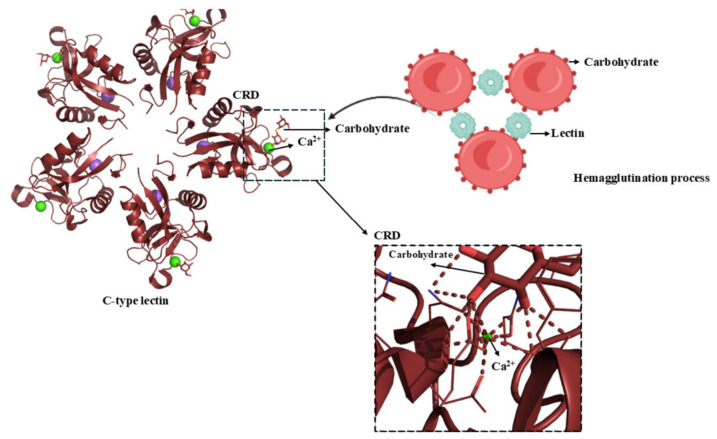
Hemagglutination process and three-dimensional structure of CTL, highlighting the CRD and Ca^2+^ interaction with the galactose carbohydrate (PDB ID: 1MUQ). Image generated using PyMOL 3.1.8 and Biorender. Brown: Secondary structure of CTLs, Purple: Sodium ions, Green: Calcium ions.

True CTLs are prevalent in approximately 69% of snake species, with a notable abundance in Viperidae [1]. The CRD typically features a negatively charged concave surface that coordinates Ca^2+^ ions to facilitate ligand stabilization [41]. Structurally, the CRD consists of a conserved fold of 110–130 residues organized into two α-helices and seven antiparallel β-sheets [4]. A functional loop situated above the beta-strands coordinates up to four Ca^2+^ ions, which are essential for both stabilizing the three-dimensional architecture and mediating carbohydrate docking [42], as illustrated in Figure 1.

**Figure 2:**
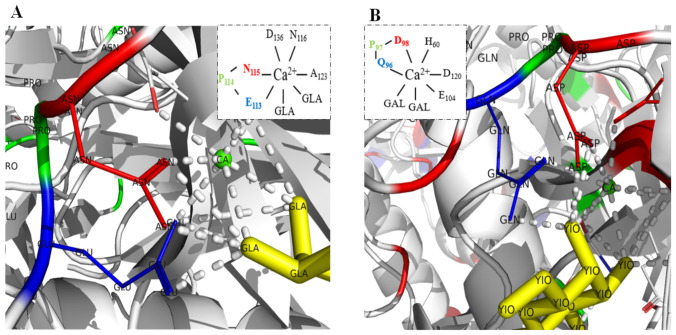
Three-dimensional representation showing the residues composing the CRD motifs and their interaction with the Ca^2+^. (**A**) EPN motif (PDB ID: 3ALU) and (**B**) QPD motif (PDB ID: 1MUQ) visualized using PyMOL 3.1.8. EPN motif are highlighted in blue (Glu), green (Pro) and red (Asn); QPD motif are highlighted in blue (Gln), green (Pro) and red (Asp).

CTLs share a conservative region of residues, which maintains a pattern of folds and disulfide bridges with about 110 to 130 residues, arranged as two α-helices and seven antiparallel β-sheets [4]. Notably, the presence of a loop located above the two β-strands represents a structurally and functionally significant aspect of Ca^2+^ interactions. Thus, CTLs can bind up to four Ca^2+^ ions, thereby contributing to carbohydrate binding and stabilizing their three-dimensional structure [42], as shown in Figure 2.

**Figure 3:**
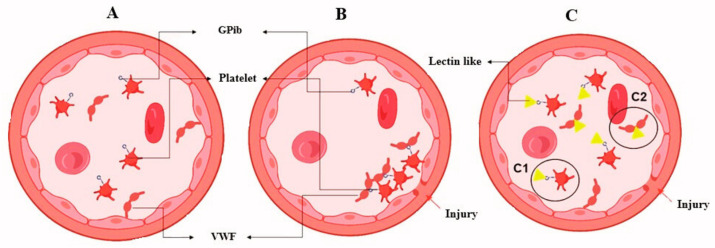
Mechanism of action of snaclecs. (**A**) Uninjured endothelial cells. (**B**) Injured endothelial cells and normal plaquetary aggregation. (**C**) Mechanisms of action of snaclec binding to VWF and GPlb, preventing platelet binding and subsequent thrombus formation. (Snaclecs are highlighted in yellow, platelets in red and erythrocytes in pink).

Specific bonds may occur upon the presence of different carbohydrate types of mannose, fructose, glucose, or galactose, depending on the binding motif. The snake C-type lectins (snaclecs) can feature two types of motifs: EPN motif (Glu-Pro-Asn) (Figure 3A), characterized by a proline residue between glutamate and aspartate residues, able to interact with D-mannose, D-glucose, L-fructose, and N-acetyl-D-glucosamine, and QPD motif (Gln-Pro-Asp) (Figure 3B) containing a proline residue between glutamine and aspartate residues, binding to D-galactose or N-acetyl-D-galactosamine [4,8,42]. Furthermore, some studies classify snaclecs by the presence or absence of the motif, even those with no motif for an interaction with Ca^2+^ [42,43,44]. Therefore, bonds between motif groups and carbohydrates occur due to a carbonyl (C=O) group within amino acid residues and hydroxyl groups (-OH) present in carbohydrates [42]. Beyond monosaccharides, CTLs facilitate complex interactions with oligosaccharides and N-glycans. This specificity is primarily governed by conserved tripeptide motifs within the CRD, such as EPN (mannose-type) or QPD (galactose-type), which coordinate with specific carbohydrate residues through hydrogen bonding and Ca^2+^ coordination [45]. Some studies suggest that different snaclecs exhibit a role in homeostasis; nevertheless, some researchers attribute heterologous biological activities, such as antibacterial, anti-biofilm, and antitumor activities [46,47,48]. Therefore, Table 1 highlights some CTLs whose heterologous activities have been identified over the past five years, emphasizing characteristics such as molecular weight, ion binding, carbohydrate binding, and biological activity.

BjcuL, a CTL isolated from *B. jararacuss* emphasizing its dependence on divalent ions (Ca^2+^ and Na^+^), specificity for galactose-containing carbohydrates, and its diverse heterologous biological activities [49]. Functionally, BjcuL exhibits both antiapoptotic and anti-inflammatory activities, reflecting a context-dependent modulatory role rather than direct cytotoxicity [39] besides modulating the immune response by releasing cytokines such as TNF-α, IL-6, and IL-10 [50]. Moreover, the effects on endothelial cells reinforce vascular and inflammatory regulation [51]. Overall, the results highlight the biotechnological potential of BjcuL as a multifunctional molecule with applications in immunomodulation, inflammation regulation, and therapeutic strategies targeting glycans.

### 3.2. C-Type Lectin-like Proteins

While sharing a common evolutionary scaffold with canonical lectins, snaclecs represent a specialized adaptation, where the ancestral carbohydrate-binding function has been replaced by high-affinity protein–protein interactions. Despite their structural homology, snaclecs exhibit distinct molecular features: they typically form heterodimers composed of highly homologous subunits and lack the conserved residues required for Ca^2+^ coordination, such as the EPN/QPD motifs. Consequently, snaclecs do not require Ca^2+^ for structural stability and have evolved to target specific protein receptors, particularly those involved in platelet aggregation and the coagulation cascade, rather than recognizing carbohydrate moieties [4,52].

The effects of CTLs and snaclecs are closely related to the homeostasis of the organism, where CTLs participate in acting directly on blood coagulation and plaquetary receptors. Accordingly, snaclecs may also affect plaquetary functions by either promoting or inhibiting, depending on their interaction with several modulators, namely von Willebrand factor (VWF), GPIb, integrin α2β1, C-type lectin-like receptor 2 (CLEC-2), and GLVI [53]. Some studies indicate that snaclecs promote the binding of VWF to GLPIb, thereby enabling plaquetary aggregation or the agglutination process. VWF and GLPIb are high molecular weight glycoproteins formed by distinct subunits that contain binding domains for certain ligands, such as collagen, and membrane receptors, including GPIb, being important for blood coagulation, especially for plaquetary tamponade. Hence, VWF is released during inflammatory processes to normalize homeostasis of the body [53], as illustrated in Figure 3.

However, snaclecs, including botrocetin isolated from *B. jararaca*, degrades VWF, resulting in thrombocytopenia observed in snake bites, caused by the venom. In contrast, brotocetin might exhibit therapeutic potential to treat thrombosis [53]. Furthermore, the α2β1 integrin is a heterodimer acting as a binding site for certain molecules, including collagen, participating in several important metabolic processes, as well as in cartilage formation.

Rodocetin, isolated from the snake *Calloselasma rhodostoma*, may act as an α2β1 integrin inhibitor by binding to an α2 domain, preventing collagen attachment and consequently impairing plaquetary aggregation via this pathway [4]. Rodocetin has been reported to interact with other receptors, such as the C-type lectin-like receptor 2 (CLEC-2), involved in plaquetary activation pathways. Moreover, metastatic dissemination in tumor biology has been linked to CLEC-2–mediated signaling, which contributes to this process through its role in promoting angiogenesis and facilitating platelet–tumor cell interactions [54].

Host responses to pathogen-associated and danger-associated molecular patterns are key components of inflammasomes. In this context, CTLs exhibit function as essential multiprotein platforms of the innate immune system, both in priming and activation phases of inflammasome signaling. Furthermore, some CTLs also contribute to the production of reactive oxygen species (ROS), which are essential for the activation of the multiprotein NLRP3 inflammasome complex. Another CLT, Convulxin (CVX), isolated from *C. durissus terrificus* venom, has been shown to interact with the Dectin-2 receptor, resulting in ROS production and subsequent activation of the multiprotein complex NLRP3 inflammasome [16]. Likewise, ROS generation and consequent multiprotein NLRP3 inflammasome complex activation are promoted by BjcuL, a lectin derived from *B. jararacussu* [37]. The functional versatility of CTLs and snaclecs is further evidenced by their interaction with a diverse array of receptors. For instance, DC-SIGN plays a pivotal role in recognizing viral and bacterial pathogens, while dectin-1 is essential for detecting fungal infections. The ability of these lectins to modulate such distinct pathways underscores their critical importance in coordinating innate immune responses [55].

Therefore, their broad functional diversity is intrinsically associated with their participation in essential physiological and pathological processes, reinforcing their relevance as promising therapeutic targets and biotechnological tools in the treatment of infectious diseases, cardiovascular disorders, and cancer [38,56]. Recently, increasing research attention and effort have been dedicated to investigating the biological applications of these molecules.

Thus far, several snaclecs have been described and fully characterized based on their key biochemical and functional properties, including ion dependence and intrinsic or heterologous biological activity, as shown in Table 2.

This highlights the functional diversity of snake venom snaclecs, particularly in their modulation of hemostasis. Many snaclecs, including bitiscetin-3, baltetin, botrocetin, rhodocytin, and protrocetin, exhibit plaquetary-related activities, either promoting or inhibiting aggregation. Furthermore, dual behavior has also been reported for afibatide and promucetin, reflecting their potential to interact with distinct plaquetary receptors and coagulation factors. Previous studies have demonstrated that snaclecs modulate key components of the hemostatic system, influencing thrombus formation and vascular homeostasis [4,64].

Beyond their hemostatic functions, some snaclecs exhibit broader pharmacological activities, including anti-inflammatory and analgesic effects. Notably, convulxin and lebecetin demonstrate similar properties [16,62]. Overall, research continues to support the growing evidence that snaclecs can modulate immune-related pathways, thereby expanding their biological relevance beyond coagulation [65]. Regardless of their variability in function, most snaclecs exhibit a conserved molecular weight range of 25–32 kilodaltons (kDa), suggesting structural conservation within this family. Thus, overall, their features reinforce the potential of snaclecs as promising scaffolds for developing drugs that target cardiovascular, inflammatory, and neoplastic disorders [16,38].

## 4. Biological Application of C-Type Lectins and Lectin-like

### 4.1. Antitumor and Antiangiogenic Activity

Many snake venom proteins have been observed to have antitumor effects, including phospholipase A2 and L-amino acid oxidase [66,67,68]. Similar activity related to angiogenesis has often been reported in CTLs, due to their ability to bind with free carbohydrates associated with membranes and other cellular structures, as well as the extracellular matrix and its components [14]. Angiogenic properties refer to the ability to induce the formation of new blood vessels from existing ones. Inducing angiogenesis is essential for several physiological processes, including healing, tissue repair, and metastasis [14]. A recent study investigated whether BjcuL induces apoptosis through the TRAIL-type death receptor by examining Lectin activity against colorectal cancer cell lines HT29 and HCT116 [69].

As previously reported, BjcuL induces the release of FADD and caspase-8, as well as other anti-apoptotic proteins such as c-FLIP and polyubiquitinated proteins, whilst apoptosis by this pathway was not observed [39]. In 2020, Carneiro-Goetten and collaborators investigated the impact of BjcuL on the migration and invasion potential of treated neuroblastoma cells, with a particular focus on neutrophil modulation. Their findings revealed that neutrophils treated with the lectin could reduce tumor cell invasion by approximately 30% compared to the control group [70].

### 4.2. Antibacterial Activity

Antibacterial activity has been reported for some CTLs isolated from snake venom, particularly against Gram-positive bacteria; by binding to carbohydrates, these lectins likely interact with the peptidoglycan present in the bacterial cell wall [47]. A study on a lectin isolated from the venom of *Bothrops oligolepis* demonstrated antibacterial activity with an MIC of 100 µg mL^−1^ against *Staphylococcus aureus*; however, the study did not report activity against Gram-negative bacteria, only against the crude venom [48]. In contrast, a lectin isolated from *B. jararacussu* snake venom did not affect the bacterial growth of S. aureus, but it showed an important reduction in the biofilm formation of the same bacterium, reducing about 50% of the biofilm at 100 µg·mL^−1^. This indicates that the lectin probably interacted with the carbohydrates present in the biofilm matrix, since it is composed of carbohydrates, like glucose and galactose, and other macromolecules [47].

### 4.3. Antithrombotic Activity

Disorders of blood coagulation and plaquetary aggregation are among the known effects of snake venoms [2]. Recently, research focusing on characterizing antiplatelet compounds derived from venom as promising candidates for antithrombotic agents intensified [71]. Arterial thrombosis, linked to conditions including heart attack, ischemic injury, and atherosclerosis, may be addressed with those agents [72]. Among the molecules present in snake venoms, some could modulate the GPIb-VWF axis, as demonstrated by CTLs [57]. The snaclecs, found in snake venoms, can affect blood clotting in different ways and have different effects [13].

Among snaclecs exhibiting antiplatelet activity, bitiscetin-3α/β (UniProt ID: A0A5A4WNG2/A0A5A4WN20) isolated from *Bitis arietans*, had plaquetary aggregation activity confirmed in the presence of VWF and GPIb, as was observed in other bitiscetin isoforms; nevertheless, studies have indicated that bitiscetin-3 inhibits VWF binding with age [57]. Furthermore, evidence suggests Bitiscetin-3 acts at the collagen recognition site of VWF and not GPIb, in contrast to other isoforms [57,73]. Similarly, anfibatide, an antithrombotic agent purified from the snake venom of *Deinagkistrodon acutus*, is described as a lectin that binds to GPIbα, where the mechanism is inhibition by competitive blockade of thrombin [59]. Preliminary in vitro studies revealed that snaclecs prevented platelet aggregation mediated by GPIbα-VWF and induced by thrombin at low doses. Moreover, anfibatide has demonstrated the potential to inhibit, adhere to, and aggregate platelets ex vivo, as well as dissolve preformed thrombi [58]. The first snaclec, whose plaquetary aggregation inhibitory activity was related to epinephrine, was described by Pereira and his collaborators, who described baltetin, isolated from *B. alternatus*, which inhibited plaquetary aggregation in a dose-dependent manner, with concentrations from 0.6 to 3 μg inhibiting 35 to 69% in 30 min of incubation [19]. Another snaclec, rhodocytin α/β (UniProt ID: Q9I841/Q9I840), isolated from the venom of the snake *Calloselasma rhodostoma*, exhibits plaquetary aggregation activation properties, unlike other snaclecs isolated from snakes that activate GPIb. Researchers observed that rhodocytin activates plaquetary aggregation using the CLEC-2 receptor [61].

### 4.4. Immunomodulation and Anti- and Pro-Inflammatory Activities

Lectins have gained recognition for their immunomodulatory properties, which involve a range of activities, including the stimulation of immunological mediators, such as pro- and anti-inflammatory responses [74]. Glycans are essential mediators of inflammatory homeostasis, functioning as molecular ‘tags’ that coordinate cellular recognition and trafficking. Specifically, the interaction between selectins and glycan ligands, such as sialylated motifs on leukocytes, governs the rolling and extravasation of immune cells into inflamed tissues [75,76].

Within this framework, C-type lectin receptors act as key checkpoints; they recognize specific glycan signatures on host cells and pathogens to trigger signaling cascades that modulate pro-inflammatory cytokines [77]. In the context of ophidian envenomation, recent glycoproteomic evidence suggests that snake venom toxins may interfere with these highly coordinated systems [78]. By mimicking or blocking these host–glycan interactions, venom CTLs and snaclecs can dysregulate immune cell recruitment and amplify the local inflammatory damage, a process that underscores their potential as both pathological agents and therapeutic targets [79].

Another reported action for snake lectins is the modulation of inflammation, both pro- and anti-inflammatory effects. Several pro- and anti-inflammatory mediators, such as tumor necrosis factor-alpha (TNF-α), interleukin-6 (IL-6), interleukin-8 (IL-8), and interleukin-10 (IL-10), contribute to the degree of the inflammatory response by initiating the healing process and removing harmful stimuli [80]. Several studies have highlighted the role of lectins in modulating the inflammatory response and tumor progression [81,82].

Lebecetin α/β (UniProt ID: P84038/P84037) isolated by *Macrovipera lebetina*, for example, has been shown to be anti-inflammatory in THP-1 macrophages because it decreases the release of pro-inflammatory cytokines, such as IL-6, IL-8, and TNF-α, while inducing the release of IL-10, which controls inflammatory processes, and some studies demonstrated that lebecetin is the first snaclec to inhibit the production of pro-inflammatory cytokines in human THP-1-derived macrophages stimulated by lipopolysaccharide (LPS) by modulating different pathways involved in the inflammatory response [38].

Researchers have demonstrated the anti-inflammatory activity of lebecetin in an in vivo murine model using formalin-induced paw edema. This effect is linked to a decrease in the release of pro-inflammatory cytokines and an increase in anti-inflammatory mediators, especially IL-10. Interestingly, greater efficacy was observed at lower doses, suggesting a biphasic hormetic response related to the modulation of inflammatory pathways. Additionally, the compound exhibited analgesic properties, as demonstrated by a significant reduction in nociceptive responses [38,62]. Accumulating evidence suggests that impeding the activation of these signaling pathways and diminishing the secretion of downstream inflammatory cytokines may be an efficacious approach to counteract the development of chronic inflammation and related diseases [83,84].

The pro-inflammatory activity of galatrox was investigated, and its capacity to activate macrophages was evaluated. Binding assays using bone marrow-derived macrophages revealed interactions with essential pattern recognition receptors, such as TLR-2, TLR-4, and Dectin-1 [38]. Macrophage activation, marked by an increased production of IL-6, TNF-α, and KC, is primarily driven by TLR-4 engagement and subsequent MyD88-dependent signaling, as demonstrated by functional data [38].

Concurrently, lectin-mediated immunomodulation has been investigated in diverse pathological contexts, including cancer. In a neuroblastoma model, the presence of BjcuL-modulated neutrophils led to a notable decrease in the invasiveness of tumor cells without impacting their migratory patterns. This observation indicates a selective control over effector functions [70]. Furthermore, lectins have been demonstrated to activate mast cells and increase vascular permeability by releasing histamine and platelet-activating factor [85].

Studies with convulxin (CVX), a C-type lectin-like protein, further support the ability of these molecules to change the immune system. In peripheral blood mononuclear cells, CVX did not promote proliferation. It induced the release of IL-10. At the same time, it did not stimulate IL-2 production. This indicates an anti-inflammatory profile associated with Dectin-2 interaction [16]. Furthermore, CVX triggered the production of reactive oxygen species in monocytes without inducing nitric oxide release; this oxidative response was linked to the activation of the NLRP3 inflammasome, which led to the secretion of IL-1β [16].

BjcuL activates the NLRP3 inflammasome in PBMCs, inducing TNF-α, IL-6, and IL-1β release via NF-κB signaling, accompanied by increased ROS production. Within one hour, it upregulates NLRP3 and IL-1β gene expression. After two hours, it increases IL-6 and caspase-1 expression. The lectin also promotes TLR4 upregulation and interaction, linking receptor activation to IL-1β production, as well as increased lactate dehydrogenase release, indicating pyroptosis after prolonged stimulation [37]. Conversely, anfibatide exhibited an anti-inflammatory profile in an ischemic stroke model by inhibiting NF-κB and NLRP3 signaling pathways, resulting in reduced pro-inflammatory cytokine production, decreased microglial activation, and attenuation of apoptotic markers such as caspase-3 [59].

## 5. Biotechnological Perspectives for C-Type Lectin

The biotechnological exploration of animal-derived compounds has transitioned from traditional screening to a data-driven era, significantly enhancing the drug discovery pipeline. A prime example of this success is the FDA-approved analgesic Ziconotide (Prialt), a synthetic peptide derived from cone snail venom, which underscores the immense pharmacological potential of venomous species [86]. Currently, the integration of high-throughput sequencing with specialized databases, such as ConoServer, the Natural Products & Biological Sources (NPBS) atlas, and dedicated lectin repositories like UniLectin3D, is essential for the rapid identification of bioactive scaffolds [87,88,89]. Furthermore, the application of deep learning and rational computational design [90,91] allows for the modification of toxin-derived proteins to overcome clinical limitations, such as toxicity and poor bioavailability, transforming once-impractical molecules into viable therapeutic candidates [92].

Peptides and proteins are already well-explored compounds within rational modification used in silico experiments, which rely on the use of software that helps predict the structure, physical–chemical characteristics, and biological application of these compounds [93]. Since about 90% of the compounds found in snake venoms are made up of this group of molecules, a contribution has been made directly to the search for a cure for various diseases [2]. CTLs are gaining traction in the scientific community, despite not being enzymatic proteins. These proteins, which make up a small percentage of venoms, possess crucial characteristics that warrant further investigation. Notably, they exhibit conserved regions in their molecular structure and display specificity for certain ligands, a property that can influence their application [1,94].

Rational design strategies for lectins have significantly evolved, transitioning from serendipitous discovery to targeted protein engineering. Recent advancements focus on modifying the CRD to fine-tune ligand specificity and affinity, particularly for identifying pathological glycans and modulating immune signaling [95,96]. As highlighted by Hombu and collaborators, contemporary protein engineering, including directed evolution and de novo computational design, allows for the development of ‘lectins by design’ with enhanced stability and tailored selectivity [97]. These engineered molecules serve as sophisticated tools for both diagnostic sensing and the development of targeted glycomimetic therapies, addressing the inherent challenges of using native toxins in clinical settings [98].

Beyond the rational design of peptides, the search for methodologies that enable the use of compounds involving snakes and their toxins has been encompassed by the use of artificial intelligence [99]. AlphaFold and ColabFold are tools that use artificial intelligence in the structural prediction of proteins, which is an important technique in the study of snake toxins, given their major protein composition [100,101]. In addition, methodologies such as these emerge to solve a problem within molecular biology that has persisted for the last 50 years regarding the prediction of protein structure [102]. In view of this, Kalogeropoulos and collaborators tested three different platforms for their ability to predict the structure of more than 1000 different types of snake proteins, whereby CTLs were analyzed, and AlphaFold was found to be the most efficient, followed by ColabFold [102]. Furthermore, artificial intelligence has risen as a promising tool for the development of novel antivenoms, as Vázquez Torres and collaborators demonstrated the design of stable proteins capable of neutralizing α-neurotoxins from Elapidae snakes, highlighting the potential of artificial intelligence to accelerate and democratize the discovery of new toxin-targeting therapeutics [103].

A peptide based on the N-terminal portion of a CTL isolated from the snake *Calloselasma rhodostoma*, also known as aggregation, was developed from a few studies that changed the molecular rationality involving CTLs. Researchers investigated the relationship between the N-terminal portion of the protein and CLEC-2 binding and observed that the fragment exhibited significant antitumor activity, making it a promising candidate for treating tumor metastasis [46]. Another smart change based on lectin was reported, when several small pieces of protein based on aggregation or rhodocytin were made and cleaned from the poison of *Calloselasma rhodostoma*, which, like vixapatin, attaches to α2β1 integrin, stopping blood cells from sticking together in tumors. The AACT peptide, derived from the C-terminal alpha chain of aggregation, demonstrated an inhibitory effect on plaquetary aggregation, anti-metastasis activity, and the prevention of tumor cell infiltration. These findings suggest its pharmacological potential in the development of anticancer drugs [46].

Furthermore, peptides derived from echicetin, a lectin extracted from *Echis carinatus*, were meticulously engineered based on its interaction with thrombin and factor Xa. Two peptides, 1A and 1B, were generated from key binding regions. Peptide 1B exhibited stronger anticoagulant activity than peptide 1A due to its ability to bind to both the heavy and light chains of thrombin and factor Xa. This results in more pronounced inhibition of these coagulation factors [104]. Therefore, the need to develop new studies involving rational molecular design and lectins is evident, especially those originating from venomous animals that present therapeutic targets.

## 6. Conclusions

In summary, while CTLs isolated from snake venoms have historically received less investigative focus than more abundant and lethally acting toxin classes, such as metalloproteinases and phospholipases A2, they represent an essential, yet under-explored, frontier in toxinology. However, the CTLs reported here show the importance of developing work that will better understand and characterize this group of proteins. Many lectins reported here have shown their effects, mostly in antitumor cells and in angiogenesis, confirming how molecules isolated from snake venom can contribute to the discovery of new treatments for several diseases that function as global public health problems, functioning as inspirations in the development of new drugs to mitigate such problems.

## Figures and Tables

**Table 1:** Molecular and functional characterization of C-type lectins isolated from *Bothrops jararacussu* venom (2020–2025).

UniProt	Ion	Lectin	Carbohydrate	Weight (KDa)	Heterologous Activity	Reference
P83519	Ca^2+^; Na^+^	BjcuL	Galactose	161.72	Antiapoptotic	[37,39]
					Anti-inflammatory	

**Table 2:** Major snaclecs isolated from snake venoms described and their main characteristics (2020–2025).

UniProt ID	Lectin/ Derivates	Species	Weight (KDa)	Heterologous Activity	References
Q7LZK5	Bitiscetin-3α	*B. arietans*	25	Plaquetary aggregation	[57]
Q7LZK8	Bitiscetin-3β				
-	Anfibatide	*D. acutus*	30	Anti-plaquetary aggregation Anti-inflammatory and anti-apoptotic	[58,59]
-	Baltetin	*B. alternatus*	25	Plaquetary Aggregation	[19]
M1V359	Botrocetin-α	*B. jararaca*	31	Antithrombotic and Platelet aggregation	[53,60]
M1VNP5	Botrocetin-β				
O93426	Convulxin-α	*C.d. terrificus*	84	Anti-inflammatory	[16]
A0A6I9UUJ4	Convulxin-β				
Q9I841	Rhodocytin-α	*C. rhodostoma*	30–32	Plaquetary aggregation	[61]
A0A0B0PKZ5	Rhodocytin-β				
C0HMC4	Protocetin-α	*P. mucrosquamatus*	29.9	Plaquetary aggregation	[21]
P84038	Lebecetin-α	*M. lebetina*	31	Anti-inflammatory and analgesic	[62]
P84037	Lebecetin-β				
-	Promucetin	*P. mucrosquamatus*	29.9	Plaquetary aggregation and anticoagulation	[20]
-	Vaa- snaclec -3/2	*V. a. ammodytes*	30	Anti-plaquetary aggregation	[63]

## Data Availability

The data presented in this study are available on request from the corresponding author.

## Supplementary Materials

The following supporting information can be downloaded at: https://www.mdpi.com/article/10.3390/molecules31111906/s1, Table S1. Primary sequences of CTLs and snaclecs retrieved from the UniProt database; Figure S1. Multiple sequence alignment of representative snake venom CTLs and snaclecs from various species.
