# Supplementary material for: Biotechnological Applications of C-Type Lectins Isolated from Snake Venoms
Source: Molecules. 2026 Jun 1;31(11):1906. doi: 10.3390/molecules31111906 (PMC13257952; doi:10.3390/molecules31111906)
Supplement: Supplementary file 1 [file molecules-31-01906-s001.zip › molecules-4262373-supplementary.pdf]

**Table S1**. Primary sequences of CTLs and snaclecs retrieved from the UniProt database. The table includes accession numbers, protein names, and the full primary sequences used in this study.

| UniProt ID | Lectin/ derivates | Sequence | Ref |
|---|---|---|---|
| | **C-type lectins** | | |
| P83519 | BJcuL | MGRFLFVASSACWFVFLSLSGAKGNNCPQD<br>WLPMNGLCYKIFNELKAWKDAEMFCRKYKP<br>GCHLASIHLYGESPEIAEYISDYHKGQSEV<br>WIGLCDKKKDFSWEWTDRSCTDYLSWDKNQ<br>PDHYQNKEFCVELVSNTGYRLWNDQVCESKN<br>AFLCQCKF | [15] |
| | **Snaclecs** | | |
| A0A5A4WNG2 | Bitiscetin-3α | MGRFIFLSSGLLAVFLSLRGTGADEGCLPD<br>WSSREGHCYKVFKEWKTWADAEKFCKELVN<br>GGHLTSFNSREEGEFIVKLAFEKMRLPSVW<br>IGLRQFWRICPLRWTDGARLDYRALSDEPI<br>CFIAKTSDNKWFQWKCSNVLGFVCKYRVPN | [16] |
| A0A5A4WN20 | Bitiscetin-3β | MGRFIFLSSGLLVVFLSLRGTGADEGCLPD<br>WSSRVEHCYKVFKERKTWEDAEKFCVENSG<br>HLASIEGKEEADFVAQLLSQALKKSKYDYN<br>VWIGLRDESKTQQCSPQWTDGSLTFYENLD<br>EPTKCFGLGEHTGYRTWTDLPCGQKNPFIC<br>KSRLPH | |
| Q8JIV9 | Anfibatide-α (Agkisacucetin) | MGRFIFVSFGLLVVFLSLSGTGADVDCLPG<br>WSAYDQSCYRVFKLLKTWDDAEKFCTERPK<br>GGHLVSIESAGERDFVAQLVSENKQTDNVW<br>LGLKIQSKGQQCSTEWTDGSSVSYENFSEY<br>QSKKCFVLEKNTGFRTWLNLNCGSEYAFVC<br>KSPP | [17] |
| Q8AYA3 | Anfibatide-β (Agkisacucetin) | MGRFIFVSFGLLVVFLSLRGTGAGFCCPLR<br>WSSYEGHCYLVVKEKKTWDDAEKFCTEQRK<br>GGHLVSVHSREEADFLVHLAYPILDLSLIW<br>MGLSNMWNDCKREWSDGTKLDFKAWAKTSD<br>CLIGKTDGDNQWLNMDCSKKHYFVCKFKL | |

| | | | |
|---|---|---|---|
| M1V359 | Botrocetin-α | MGRFIFVSFGLLVGFLSLSGTAADCPSGWS<br>SYEGNCYKFFQQKMNWADAERFCSEQAKGG<br>HLVSIKIYSREVDFVGDLVTKNIQSSDLYA<br>WIGLRVQNKEKQCSSWSDGSSVSYENVVER<br>TVKKCFALEKDLGFVLWISLYCAQKNPFVC<br>KSPPP | [19] |
| M1VNP5 | Botrocetin-β | MGRFIFVSFGLLVGFLSLSGTAADCPPDWS<br>SYEGSCYRVFEQKMDWDDAEKFCTEQQTGG<br>HLVSFQSREEADFVSSLTSPMLKADVLWIG<br>LSDVWNKCRFEWTDGMEFDYDDYYFIAEYE<br>CVASKPTNNKWWIIPCTRLKNFVCEFQA | |
| O93426 | Convulxin-α | MGRFIFVSFGLLVLFLSLSGTGAGLHCPSD<br>WYYYDQHCYRIFNEEMNWEDAEWFCTKQAK<br>GAHLVSIKSAKEADFVAWMVTQNIEESFSH<br>VSIGLRVQNKEKQCSTKWSDGSSVSYDNLL<br>DLYITKCSLLKKETGFRKWFVASCIGKIPF<br>VCKFPPQC | [20] |
| O93427 | Convulxin-β | MGRFIFVSFGLLVVFLSLSGSEAGFCCPSH<br>WSSYDRYCYKVFKQEMTWADAEKFCTQQHT<br>GSHVSFHSTEEVDFVVKMTHQSLKSTFFWI<br>GANNIWNKCNWQWSDGTKPEYKEWHEEFEC<br>LISRTFDNQWLSAPCSDTYSFVCKFEA | |
| Q9I841 | Rhodocytin-α | GLEDCDFGWSPYDQHCYQAFNEQKTWDEAE<br>KFCRAQENGAHLASIESNGEADFVSWLISQ<br>KDELADEDYVWIGLRAQNKEQQCSSEWSDG<br>SSVSYENLIDLHTKKCGALEKLTGFRKWVN<br>YYCEQMHAFVCKLLPY | [21] |
| Q9I840 | Rhodocytin-β | MGRFIFVSFGLLVVFLSLSGTGADCPSGWS<br>SYEGHCYKPFNEPKNWADAERFCKLQPKHS<br>HLVSQSAEEADFVVKLTRPRLKANLVWMGL<br>SNIWHGCNWQWSDGARLNYKDWQEQSECLA<br>FRGVHTWLNMDCSSTCSFVCKFKA | |

| | | | |
|---|---|---|---|
| - | Promucetin-α | MGRFTFVSFGLLVVFLSLSGTGADFDCIPG<br>WSAYDRYCYQAFSEPKNWEDAESFCEEGVK<br>TSHLVSIESSGEGDFVAQLVAEKIKTSFQY<br>VWIGLRIQNKEQQCRSEWSDASSVNYENLF<br>KQSSKKCYALKKGTELRTWFNVYCGRENPF<br>VCKYTPEC | [23] |
| - | Promucetin-β | MGRFIFVSFGLLVVFISLSGTEAGFCCPLG<br>WSSYDEHCYQVFQQKMNWEDAEKFCTQQHT<br>GSHLVSYESSEEVDFVVSKTLPILKASFVW<br>IGLSNVWNACRLQWSDGTELMYNAWTAESE<br>CIASKTTDNQWWSMDCSSKRYVVCKF | |
| A0A1I9KNS2 | Vaa-snaclecs-2 | MGRFISVSFGFLVMFLSLSGTGADCPSDWS<br>SYEGHCYKVFKEEMNWEDAEKFCTEQASGG<br>HLLSLKSTEEVDFMTSLVFPILKFDLIWIG<br>LSNLWRDCHWGWTDGVKLDYKAWSDKPNCY<br>VAKTIDYQWLRVDCSRTYNFICKSRVPR | [24] |
| A0A1I9KNN1 | Vaa-snaclecs-3 | MGRFISVSFGLLVVFLALSGTGADCPSDWS<br>SHEGHCYKVFRLFKTWEDAEKFCTQQVNGC<br>HLASIESVEEANFVAELVPKTLIKSKYHAW<br>IGLRDQSERQQCSSHWTDGSAVSYETVTDY<br>TKCFGLNKDKGYLEWVTLPCGDKNAFICKS<br>WIPH | |

**Figure S1.** Multiple sequence alignment of representative snake venom CTLs and snaclecs from various species. The conserved signal peptide region (N-terminal) is highlighted in yellow. Matching colors among cysteine residues indicate specific intra-chain disulfide bonds (e.g., green-to-green pairs), while the column highlighted in red represents a conserved cysteine involved into inter-chain disulfide bond. Distinct boxes explicitly delineate highly conserved motifs and domains involved in structural and functional roles (WIGL, WND, and QPD/EPN). Secondary structure elements are indicated by blue arrows (β-sheets) and red tubes (α-helices).

```
BJcuL               MGRFLFVASSACWFVFLSLSGAKG--NNCPQDWLPMNGLCYKIFNELKAWKDAEMFCRKY
Bitiscetin-3α       MGRFIFLSS-GLLAVFLSLRGTGADE-GCLPDWSSREGHCYKVFKEWKTWADAEKFCKEL
Vaa-snaclecs-2      MGRFISVSF-GFLVMFLSLSGTGA---DCPSDWSSYEGHCYKVFKEEMNWEDAEKFCTEQ
Rhodocytin-β        MGRFIFVSF-GLLVVFLSLSGTGA---DCPSGWSSYEGHCYKPFNEPKNWADAERFCKLQ
Botrocetin-β        MGRFIFVSF-GLLVGFLSLSGTAA---DCPPDWSSYEGSCYRVFEQKMDWDDAEKFCTEQ
Anfibatide-β        MGRFIFVSF-GLLVVFLSLRGTGAGF-CCPLRWSSYEGHCYLVVKEKKTWDDAEKFCTEQ
Convulxin-β         MGRFIFVSF-GLLVVFLSLSGSEAGF-CCPSHWSSYDRYCYKVFKQEMTWADAEKFCTQQ
Promucetin-β        MGRFIFVSF-GLLVVFISLSGTEAGF-CCPLGWSSYDEHCYQVFQQKMNWEDAEKFCTQQ
Bitiscetin-3β       MGRFIFLSS-GLLVVFLSLRGTGADE-GCLPDWSSRVEHCYKVFKERKTWEDAEKFCVEN
Vaa-snaclecs-3      MGRFISVSF-GLLVVFLALSGTGA---DCPSDWSSHEGHCYKVFRLFKTWEDAEKFCTQQ
Botrocetin-α        MGRFIFVSF-GLLVGFLSLSGTAA---DCPSGWSSYEGNCYKFFQQKMNWADAERFCSEQ
Convulxin-α         MGRFIFVSF-GLLVLFLSLSGTGAGL-HCPSDWYYYDQHCYRIFNEEMNWEDAEWFCTKQ
Rhodocytin-α        MGRFIFVSF-GLLVVFLSLSGTGAGLEDCDFGWSPYDQHCYQAFNEQKTWDEAEKFCRAQ
Anfibatide-α        MGRFIFVSF-GLLVVFLSLSGTGADV-DCLPGWSAYDQSCYRVFKLLKTWDDAEKFCTER
Promucetin-α        MGRFTFVSF-GLLVVFLSLSGTGADF-DCIPGWSAYDRYCYQAFSEPKNWEDAESFCEEG
                    ****  ::  .    *::* *: .    *   *      **  .     * :** **
```

```
               *: *  .    . ::                  :*       .     *:*
BJcuL          KPGCHLASIHLYGESPEI-AEYISDYHK---GQSEVWIGLCD--KKKDFSWEWTDRSCTD
Bitiscetin-3α  VNGGHLTSFNS-REEGEFIVKLAFEKMRL----PSVWIGLRQ--FWRICPLRWTDGARLD
Vaa-snaclecs-2 ASGGHLLSLKS-TEEVDFMTSLVFPILKF----DLIWIGLSN--LWRDCHWGWTDGVKLD
Rhodocytin-β   PKHSHLVS-QS-AEEADFVVKLTRPRLKA----NLVWMGLSN--IWHGCNWQWSDGARLN
Botrocetin-β   QTGGHLVSFQS-REEADFVSSLTSPMLKA----DVLWIGLSD--VWNKCRFEWTDGMEFD
Anfibatide-β   RKGGHLVSVHS-REEADFLVHLAYPILDL----SLIWMGLSN--MWNDCKREWSDGTKLD
Convulxin-β    HTGSHV-SFHS-TEEVDFVVKMTHQSLKS----TFFWIGANN--IWNKCNWQWSDGTKPE
Promucetin-β   HTGSHLVSYES-SEEVDFVVSKTLPILKA----SFVWIGLSN--WNACRLQWSDGTELM
Bitiscetin-3β  --SGHLASIEG-KEEADFVAQLLSQALKKSKYDYNVWIGLRDESKTQQCSPQWTDGSLTF
Vaa-snaclecs-3 VNGCHLASIES-VEEANFVAELVPKTLIKSK--YHAWIGLRDQSERQQCSSHWTDGSAVS
Botrocetin-α   AKGGHLVSIKIYSREVDFVGDLVTKNIQ-SSD-LYAWIGLRVQNKEKQCS-SWSDGSSVS
Convulxin-α    AKGAHLVSIKS-AKEADFVAWMVTQNIE-ESF-SHVSIGLRVQNKEKQCSTKWSDGSSVS
Rhodocytin-α   ENGAHLASIES-NGEADFVSWLISQKDELADE-DYVWIGLRAQNKEQQCSSEWSDGSSVS
Anfibatide-α   PKGGHLVSIES-AGERDFVAQLVSENKQ-T---DNVWLGLKIQSKGQQCSTEWTDGSSVS
Promucetin-α   VKTSHLVSIES-SGEGDFVAQLVAEKIK-TSF-QYVWIGLRIQNKEQQCRSEWSDASSVN
                   *: *  .    . ::                   :*       .     *:*

BJcuL          YLSWDKNQPDHYQNKEFCVELVSNTGYRLWNDQVCESKNAFLCQCKF--
Bitiscetin-3α  YRAL--SD------EPICFIAKT--SDNKWFQWKCSNVLGFVCKYRVPN
Vaa-snaclecs-2 YKAW--SD------KPNCYVAKT--IDYQWLRVDCSRTYNFICKSRVPR
Rhodocytin-β   YKDW--QE------QSECLAFR---GVHTWLNMDCSSTCSFVCKFKA--
Botrocetin-β   YDDYYFIA------EYECVASKP--TNNKWWIIPCTRLKNFVCEFQA--
Anfibatide-β   FKAW--AK------TSDCLIGKT-DGDNQWLNMDCSKKHYFVCKFKL--
Convulxin-β    YKEW--HE------EFECLISRT--FDNQWLSAPCSDTYSFVCKFEA--
Promucetin-β   YNAW--TA------ESECIASKT--TDNQWWSMDCSSKRYVVCKF----
Bitiscetin-3β  YENLDEP--------TKCFGLGEHTGYRTWTDLPCGQKNPFICKSRLPH
Vaa-snaclecs-3 YETVTDY--------TKCFGLNKDKGYLEWVTLPCGDKNAFICKSWIPH
Botrocetin-α   YENVVERT------VKKCFALEKDLGFVLWISLYCAQKNPFVCKSPPP-
Convulxin-α    YDNLLDLY------ITKCSLLKKETGFRKWFVASCIGKIPFVCKFPPQC
Rhodocytin-α   YENLIDLH------TKKCGALEKLTGFRKWVNYYCEQMHAFVCKLLPY-
Anfibatide-α   YENFSEYQ------SKKCFVLEKNTGFRTWLNLNCGSEYAFVCKSPP--
Promucetin-α   YENLFKQS------SKKCYALKKGTELRTWFNVYCGRENPFVCKYTPEC
               :                 *           *    *     .:*:
```
